# Mitogenomic phylogeny of Typhlocybinae (Hemiptera: Cicadellidae) reveals homoplasy in tribal diagnostic morphological traits

**DOI:** 10.1002/ece3.8982

**Published:** 2022-06-06

**Authors:** Bin Yan, Christopher H. Dietrich, Xiaofei Yu, Meng Jiao, Renhuai Dai, Maofa Yang

**Affiliations:** ^1^ 71206 Institute of Entomology Guizhou University Guiyang Guizhou China; ^2^ 71206 College of Tobacco Science Guizhou University Guiyang Guizhou China; ^3^ 420825 Shandong Museum Jinan Shandong China; ^4^ Illinois Natural History Survey Prairie Research Institute University of Illinois Champaign Illinois USA

**Keywords:** ancestral character state reconstruction, Auchenorrhyncha, classification, high‐throughput sequencing, Membracoidea, microleafhopper, mitochondrial genes, phylogenetic analysis

## Abstract

The subfamily Typhlocybinae is a ubiquitous, highly diverse group of mostly tiny, delicate leafhoppers. The tribal classification has long been controversial and phylogenetic methods have only recently begun to test the phylogenetic status and relationships of tribes. To shed light on the evolution of Typhlocybinae, we performed phylogenetic analyses based on 28 newly sequenced and 19 previously sequenced mitochondrial genomes representing all currently recognized tribes. The results support the monophyly of the subfamily and its sister‐group relationship to Mileewinae. The tribe Zyginellini is polyphyletic with some included genera derived independently within Typhlocybini. Ancestral character state reconstruction suggests that some morphological characters traditionally considered important for diagnosing tribes (presence/absence of ocelli, development of hind wing submarginal vein) are homoplastic. Divergence time estimates indicate that the subfamily arose during the Middle Cretaceous and that the extant tribes arose during the Late Cretaceous. Phylogenetic results support establishment of a new genus, *Subtilissimia* Yan & Yang gen. nov., with two new species, *Subtilissimia fulva* Yan & Yang sp. nov. and *Subtilissimia pellicula* Yan & Yang sp. nov.; but indicate that two previously recognized species of *Farynala* distinguished only by the direction of curvature of the processes of the aedeagus are synonyms, that is, *Farynala dextra* Yan & Yang, 2017 equals *Farynala sinistra* Yan & Yang, 2017 syn. nov. A key to tribes of Typhlocybinae is provided.

## Graphical abstract

We studied the evolutional relationships of Typhlocybinae based on 47 typhlocybine mitogenomic dataset representing all currently recognized tribes. The Ancestral character state reconstruction suggests that some morphological characters (fore‐ and hind wing patterns) traditionally considered important for diagnosing typhlocybine tribes are homoplastic. The divergence time estimates indicate that the subfamily Typhlocybinae arose during the Middle Cretaceous and that the extant tribes arose during the Late Cretaceous.

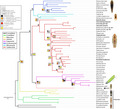

## 1 INTRODUCTION

The subfamily Typhlocybinae (Hemiptera, Membracoidea, Cicadellidae) is a large group of mostly tiny, delicate leafhoppers that feed primarily on leaf parenchymal cell contents of their host plants, thus differing from the phloem‐ or xylem‐feeding (Cicadellinae) preferences exhibited by most other leafhoppers (Bartlett et al., 2018; Dietrich, 2013a). This group is distributed worldwide and comprises numerous agricultural pests (Nault & Ammar, 1989; Sun, 2004; Wearing et al., 2011). Examples include the potato leafhopper, *Empoasca fabae*, and tea green leafhopper, *Matsumurasca onukii* (Backus et al., 2005; Chasen et al., 2014; Qin et al., 2015). However, the vast majority of species, which feed on a wide variety of plants, appear to be of little or no economic importance. The group remains poorly studied, partly because their delicate nature makes them difficult to preserve for morphological studies. Based on the large number of described species (approximately 5000), Typhlocybinae is the second largest cicadellid subfamily (after Deltocephalinae; Bartlett et al., 2018) but the actual diversity of the group is probably much higher (Dietrich, 2013a). Typhlocybines are readily distinguished from other leafhoppers by the following morphological characters: forewing fully developed without closed anteapical cells; hind tarsomere Ⅰ acuminate, without transverse row of blunt setae (Dietrich, 2005).

Since Kirschbaum (1868) first recognized the subfamily, various authors employed different concepts of Typhlocybinae and its tribes (Table S1), with as few as four and as many as eleven tribes recognized (Ahmed, 1983; Dietrich, 2013a; Dietrich et al., 2017; Dworakowska, 1979; Gebicki & Szwedo, 2006; Hamilton, 1998; Mahmood & Ahmed, 1968; Metcalf, 1968; Oman et al., 1990). Dworakowska’s (1979) six‐tribe typhlocybine classification including Alebrini, Empoascini (with synonyms Jorumini and Helionini), Erythroneurini (with synonym Bakerini), Dikraneurini (with synonym Forcipatini), Typhlocybini (with synonym Eupterygini), and Zyginellini has been followed by most recent authors (Han et al., 2020; Song, 2010; Zhang, 1990; Zhou et al., 2020). However, the status of Zyginellini has remained controversial, with recent authors regarding it either as a synonym of Typhlocybini (Ahmed, 1983; Balme, 2007; Dietrich, 2013a; Zhou et al., 2020), or as an independent and valid tribe (Dietrich et al., 2017; Lu et al., 2021; Song, 2010; Zhang, 1990).

The current tribal classification of Typhlocybinae is based almost entirely on a few characters of the wing venation. Compared with other cicadellid tribes, typhlocybines have the venation of the fore‐ and hind wings relatively reduced. Thus, particular patterns of reduction and loss or consolidation of veins have traditionally been used to define the different tribes. Alebrini, the tribe traditionally considered to be the most “primitive,” is the only tribe with an appendix on the forewing, a trait shared with non‐typhlocybine leafhoppers. Empoascini lack the section of the hind wing submarginal vein that extends along the costal margin. Erythroneurini and most Typhlocybini have the hind wing submarginal vein completely lacking at the wing apex. Erythroneurini and some Dikraneurini have hind wing vannal vein unbranched but Dikraneurini retain a complete submarginal vein.

Young (1965) transferred Mileewini (including genera *Amahuaka* and *Ujna*) into Typhlocybinae based on intuitive morphological criteria, but he later suggested that Mileewinae should be treated as a separate subfamily (Young, 1968), a classification that has been followed by most subsequent authors. Phylogenetic analyses of Membracoidea based on morphology and DNA sequence data yielded inconsistent results. The concatenated maximum likelihood (ML) analysis of anchored‐hybrid data by Dietrich et al. (2017) placed Eurymelinae (sensu lato) as sister to Typhlocybinae but with only 54% bootstrap support. An earlier morphology‐based analysis of Cicadellidae (Dietrich, 1999) placed Mileewinae (in part, Mileewini) as sister to Typhlocybinae but the first molecular phylogeny of this family (Dietrich et al., 2001) did not consistently recover Typhlocybinae as monophyletic and its relationships to other subfamilies were also inconsistently resolved. A subsequent combined analysis of morphological and 28S rDNA sequence data supported the monophyly of Typhlocybinae and its sister relationship to Mileewini (Dietrich et al., 2005). The sister‐group relationship receiving moderate bootstrap support (lower than 85%) was also recovered by Chen et al. (2021) and Lu et al. (2021).

As for most highly diverse and poorly studied insect groups, research on typhlocybine systematics continues to focus mainly on discovery and description of new taxa. Relatively few efforts have been made to assess the phylogeny of the group and test the monophyly of recognized taxa. Previous analyses have supported the monophyly of Typhlocybinae but its sister‐group has remained uncertain (Balme, 2007; Dietrich et al., 2005; Lu et al., 2021). Zhang (1990) proposed an intuitive morphology‐based hypothesis of tribal relationships within Typhlocybinae, considering Alebrini to be the earliest diverging lineage based on the retention of an appendix (shared with other leafhoppers) in the forewing, with Dikraneurini sister to Empoascini based on the relatively well‐developed submarginal vein (smv) of the hind wing. In an unpublished dissertation, Balme (2007) conducted the first explicit cladistic analysis of the group, using 73 discrete morphological characters and two molecular markers (16S rRNA and Histone 3) and proposed a classification including four tribes, Alebrini + (Typhlocybini + Empoascini) + Dikraneurini, with Erythroneurini treated as a synonym of the latter tribe. An anchored hybrid enrichment‐based phylogenomic analysis of Membracoidea based on 388 genetic loci and more than 99,000 aligned nucleotides (Dietrich et al., 2017) included only 1–2 representatives of each tribe but recovered Alebrini as sister to Empoascini and this clade sister to a clade comprising Typhlocybini and Erythroneurini + Dikraneurini with strong support. This dataset did not include a taxon sample large enough to test the monophyly of individual tribes or examine relationships among genera within tribes. Most recently, Lu et al. (2021) analyzed a much larger sample of typhlocybine taxa using data from fragments of three nuclear and two mitochondrial genes, recovering the same tribal relationships found by Dietrich et al. (2017) and also recovering Zyginellini as sister to Typhlocybini but with low support.

Here, we use sequence data from complete mitochondrial genomes in an attempt to improve resolution of phylogenetic relationships within Typhlocybinae and examine the evolution and stability of wing characters traditionally used for the classification of typhlocybine tribes. A total of 110 leafhopper mitochondrial genome assemblies have been previously deposited in GenBank. Among them, only 19 typhlocybine species are included representing the tribes Typhlocybini, Empoascini, Erythroneurini, and Zyginellini. Data for species of Alebrini and Dikraneurini were not previously available. Therefore, prior to our study, mitogenome data for Typhlocybinae remained insufficient to facilitate a comprehensive phylogenetic analysis of the subfamily.

For this study, we assembled and annotated 28 new typhlocybine mitogenomes using next‐generation sequencing (NGS) data, and conducted a comprehensive phylogenetic analysis including 19 previously available mitogenomes, representing all currently recognized tribes, to examine relationships among major lineages of the subfamily. We performed ancestral character state reconstruction (ACSR) to examine the evolution of key morphological characters. We also used molecular divergence time methods to estimate the times of origin of various typhlocybine clades.

## 2 MATERIALS AND METHODS

### 2.1 Taxon sampling and DNA extraction

In this study, single individuals of 28 typhlocybine species were newly sequenced, including one representative of Alebrini, three from Dikraneurini, three from Zyginellini, and 21 from Typhlocybini. Vouchers are deposited at the Institute of Entomology, Guizhou University, Guiyang, China (GUGC). Nineteen additional mitogenome sequences were obtained from GenBank. The 47 mitogenomes cover all currently recognized typhlocybine tribes (*sensu* Dworakowska, 1979). Based on results of prior analyses of Membracoidea, we selected representatives of Mileewinae (three species), Cicadellinae (five species) and Evacanthinae (two species) as outgroups (Balme, 2007; Dietrich, 2013b; Dietrich et al., 2010; Takiya, 2007). Included taxa including voucher number, GenBank accession number, and collection locality are listed in Table S2. All specimens were identified to species before DNA extraction. Genomic DNA was extracted from the legs and from the thoracic muscle tissue using the DNeasy Blood and Tissue kit (Qiagen, Germany) following the animal tissue protocol.

### 2.2 Mitogenome sequencing, assembly, and annotation

Illumina TruSeq libraries were prepared with an average insert size of 300 bp and sequenced on the Illumina NovaSeq 6000 platform (Beijing Berry Bioinformatics Technology Co., Ltd, China) generating 150 bp paired‐end reads. The mitochondrial genome of typhlocybine species was assembled with NOVOPlasty v2.7.0 (Dierckxsens et al., 2017), using COI sequences (MN661136, MN699874, MG397188, KY039138, MT488436 and NC_046037) as seeds and the K‐mer value set to 39. Annotation and visualization of the mitogenomes was accomplished with MitoZ v1.04 (Meng et al., 2019). Mitogenome sequences with low‐quality assembly results were submitted to MITOS WebServer (Bernt et al., 2013), under default settings and the invertebrate genetic code. Gene boundaries were defined using the ARWEN v1.2 (Laslett & Canbäck, 2008), and Geneious Prime (Kearse et al., 2012). The 28 newly assembled mitogenome sequences were deposited in GenBank (Table S2).

### 2.3 Phylogenetic analyses

The nucleotide sequences of 13 protein‐coding genes (PCGs) and two ribosome rRNAs (12S rRNA + 16S rRNA) and amino acids (13 PCGs) were aligned using MAFFT v7.394 (Katoh & Standley, 2013) with the highly accurate L‐INS‐I strategy, trimmed using trimAl v1.4.1 (Capella‐Gutiérrez et al., 2009) with the heuristic method “automated1” to remove gap‐only and ambiguous‐only positions, and concatenated using FASconCAT‐G v1.04 (Kück & Longo, 2014). Finally, we generated three matrices for the tree inference: (1) amino acids sequence with the 13 protein‐coding genes (PCGs_faa); (2) nucleotide sequence of 13 protein code genes with the third codon excluded (PCG12_fna); (3) nucleotide sequence of PCG12_fna plus the two ribosomal RNAs (PCG12_fna plus two rRNAs). Third codon positions were excluded from the nucleotide‐based analyses to reduce the possibility of bias or long‐branch attraction due to substitution saturation among species belonging to different genera (Leebens‐Mack et al., 2005; Stefanović et al., 2004). We used both partitioned and nonpartitioned approaches for phylogenetic inference. Partitioned maximum likelihood reconstructions were performed using IQ‐TREE v1.6.3 (Nguyen et al., 2015) with 1000 ultrafast bootstrap (UFBoot; Hoang et al., 2018) and 1000 SH‒aLRT replicates (Guindon et al., 2010). The best partitioning scheme and substitution models were selected based on MODELFINDER (Kalyaanamoorthy et al., 2017) implemented in IQ‐TREE. Nonpartitioned reconstructions were made using site heterogeneous models in both maximum likelihood (ML) and Bayesian inference (BI). Posterior mean site frequency (PMSF) model (Wang et al., 2018) was used for the PCGs_faa matrix by specifying a profile mixture model with the option “‐mtInv+C60+FO+R” in IQ‐TREE. The corresponding partitioned tree (PCGs_faa matrix) was treated as an initial guide tree. Bayesian inference using PhyloBayes MPI v1.8b (Lartillot et al., 2013) was performed for the PCGs_faa matrix as well. Two separate chains were independently run for 10,000 generations under the CAT+GTR model (Lartillot & Philippe, 2004) using a starting tree derived from PMSF ML analyses. We used the program bpcomp (maxdiff value) and tracecomp (minimum effective size) to check for convergence, that is, when the maxdiff value is smaller than 0.3 and minimum effective sizes are larger than 50. In discussing branch support, we consider values greater than 98% (SH‐aLRT, UFBoot2) and 0.99 (posterior probability) represent “high” support; values of 80%–98% for SH‐aLRT, 95%–98% for UFBoot2 and 0.95–0.99 for posterior probability indicate “moderate” support; and values lower than 79% for SH‐aLRT, 95% for UFBoot2 and 0.95 for posterior probability are “low” support. Pairwise distances (*p*‐distance) based on the nucleotide sequence of 13 PCGs are shown in Table S3.

### 2.4 Tree topology comparison

Three aspects of typhlocybine phylogeny that have been previously considered controversial were tested under a likelihood theory framework: (a) the sister‐group relationship (Cicadellinae, Typhlocybinae); (b) the sister‐group relationship (Typhlocybini, Zyginellini); (c) ((Empoascini, Alebrini), (Typhlocybini, (Erythroneurini, Dikraneurini))). Approximately unbiased (AU) (Shimodaira, 2002), bootstrap proportion (BP; Kishino et al., 1990), expected likelihood weight (ELW; Strimmer & Rambaut, 2002), Kishino‐Hasegawa (KH) test (Kishino & Hasegawa, 1989), Shimodaira‐Hasegawa (SH; Shimodaira & Hasegawa, 1999), Weighted KH (WKH), and weighted SH (WSH) tests were performed in IQ‐TREE v.1.6.3 with the options: ‐au, ‐zb, and ‐zw. The number of RELL replicates was specified as 10,000. Probability values (*p*‐value) of the AU test smaller than .05 indicate that the hypothesis was rejected. In cases where these statistical tests failed to reject either alternative topology, we performed four‐cluster likelihood mapping (FcLM; Minh et al., 2020; Schmidt et al., 2002) to further validate the result.

### 2.5 Ancestral character state reconstruction

Ancestral states were reconstructed for the following eight morphological characters that have been used previously to infer evolutionary trends within the group and to define tribes: (a) ocelli (absent or present); (b) forewing appendix (absent or present); (c) forewing closed anteapical cells (absent or present); (d) hind wing anal vein (branched or unbranched); (e) hind wing with distal extension of CuA vein beyond submarginal vein (beyond submarginal vein or not); (f) hind wing CuA vein (bifurcated near apex or not); (g) hind wing RP and MA vein (connected by cross vein or confluent); (h) hind wing submarginal vein apparently connected directly to CuA vein (absent or present). The eight characters were coded for terminal (tip) taxa as shown in Table S4.

The condition of character f is uncertain for species previously placed in Zyglinellini because the curved vein connected below the main stem of CuA could be interpreted either as a branch of CuA or as part of the submarginal vein, so this character was scored as uncertain (?) for the 6 included Zyglinellini species (Table S4). The description of each character and its states followed Dietrich (2013a) and Dworakowska (1993). Ancestral character state reconstruction (ACSR) was performed with a maximum likelihood approach using a single‐rate Mk1 model in MESQUITE v3.20 (Maddison & Maddison, 2017). To account for phylogenetic uncertainty, we traced character history on 10,000 sampled posterior Bayesian trees and summarized results on the BI consensus tree. In addition, we also traced character history and summarized results on the ML tree based on the matrix of the PCG12_fna.

### 2.6 Divergence time estimates

The MCMCTREE program of PAML v4.9 package (Yang, 2007) was used to estimate divergence times with the approximate likelihood calculation method based on five calibration points, four of which were based on fossils used previously to calibrate nodes in the broader analysis of Membracoidea by Dietrich et al. (2017; Table 1). Because fossil leafhoppers have not yet been incorporated into explicit morphology‐based phylogenetic analyses, we used available fossils to calibrate the root nodes of their respective tribes in order to provide conservative estimates of the minimum ages of these groups. Based on the previous molecular timetree of Membracoidea (Dietrich et al., 2017) and information on the oldest Membracoidea included in the Paleobiology Database (PDBD) Navigator website (http://paleobiodb.org/navigator/), the maximum age of the root node was constrained with a relaxed lower bound of 174.1 million years ago (MYA). The fossil species *Jassoqualus hispaniolensis* from Oligo‐Miocene Dominican amber and *Youngeawea bicolorata* from Eocene Baltic amber were used to provide minimum age calibration points for Evacanthinae and Mileewinae, respectively. Unidentified fossil species of the tribes of Cicadellini sp. and Dikraneurini sp. from Dominican amber were used to provide minimum age calibration points for Cicadellini and Dikraneurini, respectively (Table 1). The input tree was retrieved from the PMSF topology. Two priors for the rgene gamma and sigma 2 gamma were set to (2, 20, 1) and (1, 10, 1), respectively. We used an independent rates model and alpha of 0.5. The first 10^4^ iterations were discarded as burn‐in, and 10,000 samples were sampled every 5 iterations.

**TABLE 1:** Fossil taxa used for node calibration

Node no.	Fossil taxa	Current placement	Age estimate	Source	Citation
a	N/A	N/A	<174.1 MYA	N/A	Dietrich et al. (2017)
b	*Jassoqualus hispaniolensis*	Evacanthinae: Nirvanini	>17.5<110 MYA	Oligo‐Miocene Dominican amber	Dietrich and Vega (1995)
c	*Youngeawea bicolorata*	Mileewinae: Mileewini	>44 MYA	Eocene Baltic amber	Gebicki and Szwedo (2001)
d	Cicadellini gen? sp?	Cicadellinae: Cicadellini	>17.5<90 MYA	Dominican amber	Dietrich and Vega (1995)
e	Dikraneurini gen. sp?	Typhlocybinae: Dikraneurini	>17.5<90 MYA	Dominican amber	Dietrich and Vega (1995)

Note

The “N/A” indicates not applicable.

### 2.7 Morphology

The length of the body reported in the descriptions includes the forewings at rest. Male genitalia were prepared using the techniques described by Oman (1949). Morphological terminology follows Dietrich (2005, 2011) and Dworakowska (1993). The genital segments of the specimens examined were macerated in 10% NaOH for 7–10 h (or boiled for 1–3 min), then transferred to glycerin after rinsing in distilled water to remove traces of NaOH (10%) for further research. Male genitalia were drawn using a LEICA m125 microscope and drawings were processed using Adobe Illustrator CS6. Habitus photographs were taken with a KEYENCE VHX‐6000 digital camera and optimized with Adobe Photoshop CS6. All specimens studied are housed in the Institute of Entomology, Guizhou University, Guiyang, China (GUGC).

## 3 RESULTS

### 3.1 Mitogenome features

**FIGURE 1:**
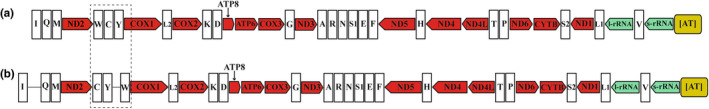
Schematic diagram of typhlocybine mitochondrial genome. a, the usual type of mitogenome in Typhlocybinae; b, the type of *Shaddai* sp

Newly sequenced mitochondrial genomes of 28 species representing 21 genera in the subfamily Typhlocybinae ranged from 14,396 bp to 16,931 bp in length, including 37 typical insect mitochondrial genes and a control region (Table S2). Mitochondrial gene arrangement is highly conservative and consistent with the hypothetical ancestral insect (Figure 1a), except in the newly sequenced *Shaddai* sp. (GenBank, MW284820), which has the gene trnW translocated behind trnY, with 691 bp and 460 bp intergenic spacers between trnI and trnQ, trnY and trnW, respectively (dashed line box in Figure 1b). This is the first report of a mitochondrial gene rearrangement within this subfamily.

### 3.2 Phylogeny

**FIGURE 2:**
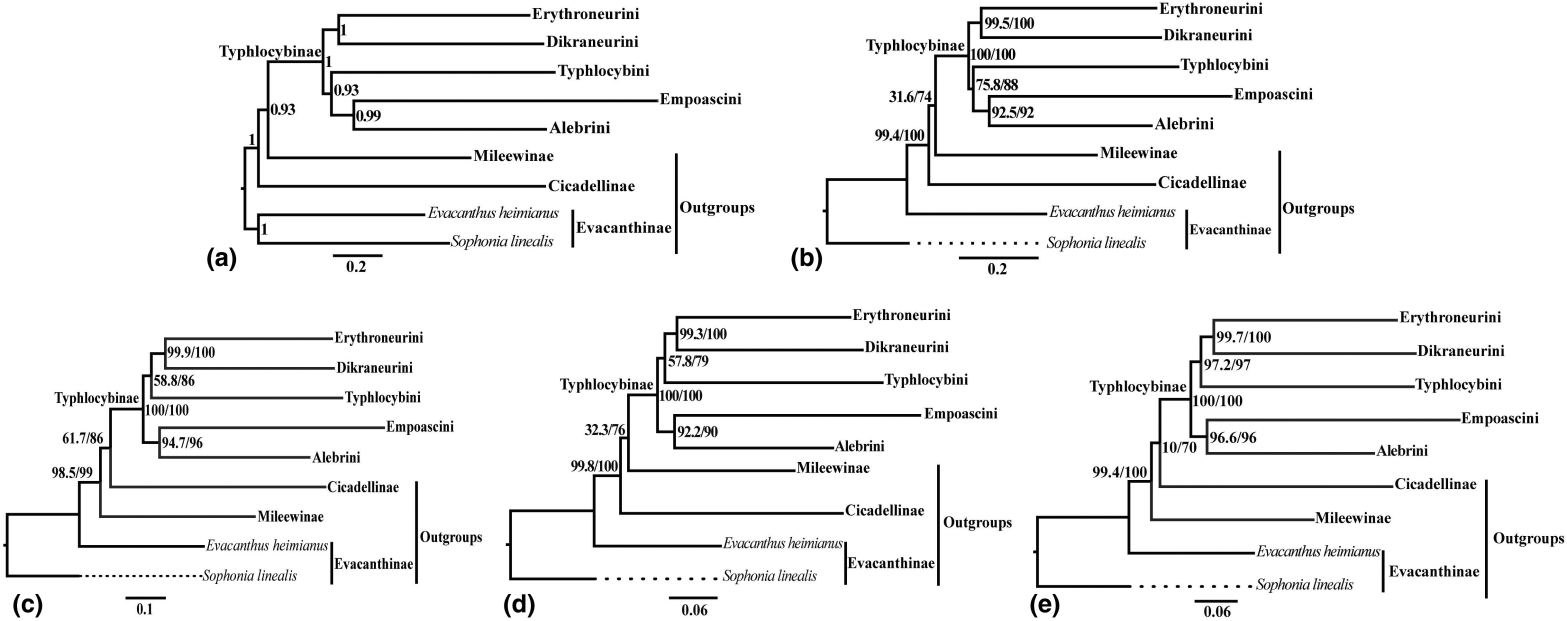
Phylogenetic relationships of Typhlocybinae reconstructed with maximum likelihood (ML) and Bayesian inference (BI) from different mitochondrial datasets. A, BI tree (CAT+GTR model), using amino acid sequence of PCGs; B, ML tree (PMSF model), using amino acid sequence of PCGs; C, ML tree (Partitioning model), using amino acid sequence of PCGs; D, ML tree (Partitioning model), using nucleotide sequence of PCGs_12; E, ML tree (Partitioning model), using nucleotide sequence of PCGs_12 concatenated 2 rRNAs

**FIGURE 3:**
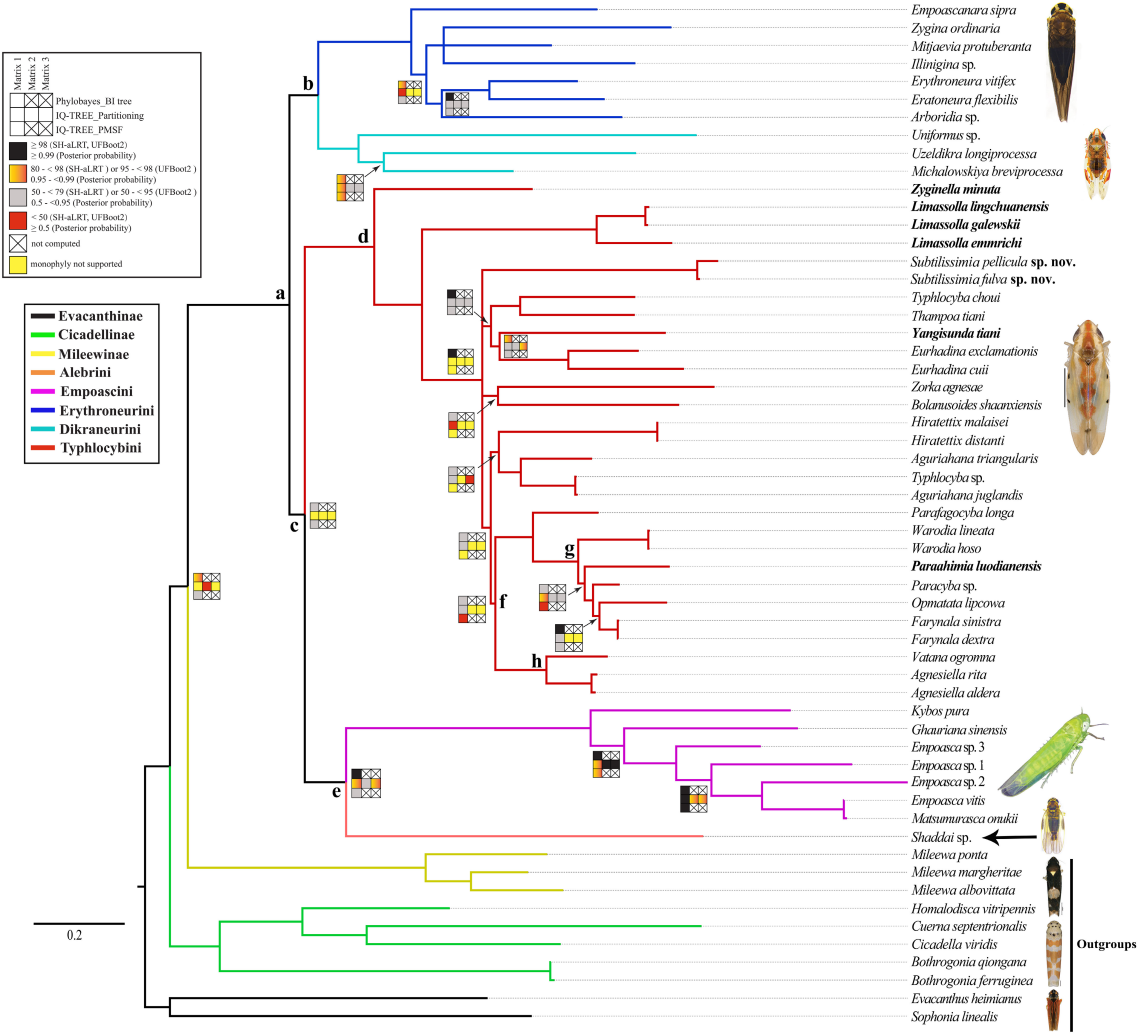
Phylogenetic tree based on the PhyloBayes analysis of amino acid matrix with the site‐heterogeneous CAT+GTR model. Node values for all analyses are plotted on or below respective clades as specified in the legend in the top‐left corner, except those with consistently high support values (PP≥0.99, SH‒aLRT & UFB≥98). Branches are colored matching the color chart on the left. For more information of trees, refer to figures S1–S5. Taxa previously included in Zyginellini are indicated in bold

The aligned amino acid dataset (PCGs_faa) included 3,467 sites. The aligned nucleotide sequence datasets included 6934 sites (PCG12_fna) and 9218 sites (PCG12_fna+2rRNAs), respectively. The character partitions and models used in the partitioned analyses are shown in Table S5. A total of five phylogenetic trees were generated, including one Bayesian inference tree (maxdiff value = 0.11, minimum effective sizes >58; Figure S1) and four maximum likelihood trees (Figures S2–S5). Overall, the trees resulting from different analyses are similar, with areas of incongruence limited to branches that received only low to moderate support in one or more analysis. The BI tree was selected as the preferred topology for subsequent analyses. As expected, all phylogenetic analyses robustly supported the monophyly of Typhlocybinae with high nodal support values (PP = 1, SH‒aLRT = 100, UFB = 100; Figure 2). Unlike the ML trees from concatenated, partitioned amino acid and nucleotide sequences of PCGs and rRNAs (Figure 2c,e), which support sister‐group relationship of Cicadellinae and Typhlocybinae, the other three phylogenetic trees consistently support Mileewinae (Mileewini) as sister to Typhlocybinae (Figure 2a,b,d). However, these alternative phylogenetic arrangements received low to moderate nodal support. Within Typhlocybinae, the sister‐group pairs Dikraneurini+Erythroneurini (Figure 3b) and Alebrini+Empoascini (Figure 3e) were recovered with moderate to strong nodal support. However, the results differed in the placement of Typhlocybini, with the BI tree (CAT+GTR model) and ML tree (PMSF model) placing this tribe sister to Alebrini+Empoascini and other three ML trees (partitioning model) placing Typhlocybini sister to Dikraneurini+Erythroneurini. The included representatives of Zyginellini (Figure 3, in bold) were never recovered as a monophyletic group but were derived from Typhlocybini, consistent with the five tribe classifications adopted by some previous authors (Ahmed, 1983; Dietrich, 2013a; Zhou et al., 2020). *Zyginella*, the type genus of Zyginellini, and *Limassolla* were consistently placed as sister to the rest of Typhlocybini with strong nodal support, but other included genera of Zyginellini (*Yangisunda* and *Paraahimia*) are deeply nested within Typhlocybini with low to moderate nodal support (Figure 3). Several additional internal nodes within Typhlocybini were also unstable (Figures S1–S5).

Statistical tests of alternative tree topologies rejected hypotheses a (sister‐group relationship of Cicadellinae and Typhlocybinae) and b (sister‐group relationship of Typhlocybini and Zyginellini) but failed to reject either alternative hypothesis of the relationship of Typhlocybini to the other tribes (Table 2). Four‐cluster likelihood mapping of the latter relationship (results not shown) also yielded equivocal results. Thus, the placement of Typhlocybini must be considered equivocal according to our results. The recent analysis of Lu et al. (2021) based on sequence data from five genes recovered Typhlocybini as sister to Dikraneurini+Erythroneurini but with only low to moderate nodal support. All analyses consistently supported the monophyly of the four tribes for which more than one exemplar was included: Dikraneurini, Empoascini, Erythroneurini, Typhlocybini (sensu lato, including Zyginellini). Zyginellini was consistently polyphyletic with the four included genera each representing an independent branch (Figure 3). Many branches pertaining to relationships within tribes were consistently well supported in all analyses but some were unresolved, particularly within Typhlocybini; for example, relationships among four major lineages of Typhlocybini were resolved inconsistently across analyses and form a polytomy in the Bayesian consensus tree (Figure 3).

**TABLE 2:** Statistical tests of alternative tree topology hypotheses conducted by IQ‒TREE v.1.6.3. Monophyly constraints: a, sister‐group relationship of Cicadellinae to Typhlocybinae; b, sister‐group relationship (and reciprocal monophyly) of Typhlocybini and Zyginellini; c, alternative tribal topology recovered by some analyses ((Empoascini, Alebrini), (Typhlocybini, (Erythroneurini, Dikraneurini)))

Hypothesis	log L	deltaL	bp‐RELL	*p*‐KH	*p*‐SH	*p*‐WKH	*p*‐WSH	c‐ELW	*p*‐AU
a	–127,274.68	13898	0–	0–	0–	0–	0–	0–	2.2e–78–
b	–115,531.50	2154.4	0–	0–	0–	0–	0–	0–	8.7e–06–
c	–113,406.30	29.17	.11+	.11+	.70+	.11+	0.27+	0.11+	0.10+

Note

The value of deltaL indicates that logL differs from the maximal log1 in the comparison. bp‐RELL, bootstrap proportion using RELL method (Kalyaanamoorthy et al., 2017; Kishino et al., 1990); *p*‐KH, *p*‐value of one sided Kishino‐Hasegawa test (Nguyen et al., 2015); *p*‐SH, *p*‐value of Shimodaira‐Hasegawa test (Kishino et al., 1990); c‐ELW, Expected Likelihood Weight (Hoang et al., 2018). *p*‐AU, *p*‐value of approximately unbiased (AU) test (Wang et al., 2018). Plus signs denote the 95% confidence sets. Minus signs denote significant exclusion. All test performed on 1000 replicates using the RELL method.

The phylogenetic results, combined with evidence from morphological characters (see below) and genetic distances (Table S3) support the establishment of a new genus, *Subtilissimia* Yan & Yang gen. nov., including two new species: *Subtilissimia fulva* Yan & Yang sp. nov. and *Subtilissimia pellicula* Yan & Yang sp. nov., but suggest that the species *Farynala sinistra* Yan & Yang, 2017 and *Farynala dextra* Yan & Yang, 2017, which have the male aedeagus identical but with processes curved in opposite directions (Yan & Yang, 2017, figures 13–32), should be treated as synonyms (*p*‐distance < .7%, except COX3 gene), *Farynala sinistra* Yan & Yang, 2017 syn. nov. The previously sequenced species identified as “*Typhlocyba* sp.” (GenBank, KY039138) belongs to the genus *Aguriahana* Distant, 1918 (COX1 *p*‐distance = 1.2%; PP = 1, SH‒aLRT = 100, UFB = 100). Based on previous studies (Qin et al., 2015; Yu et al., 2015) and analyses of phylogeny and genetic distance, we also show that the species previously identified as “*Empoasca vitis* (GenBank, NC_024838)” probably equals *Matsumurasca onukii* (COX1 *p*‐distance = .3%). Other recent studies have shown that the latter species, a major pest of tea, has been widely misidentified in China (Qin et al., 2014, 2015; Yu et al., 2015). Unfortunately, we were unable to check voucher specimens from previous studies in order to confirm the identifications suggested by our results.

### 3.3 Ancestral character state reconstruction

**FIGURE 4:**
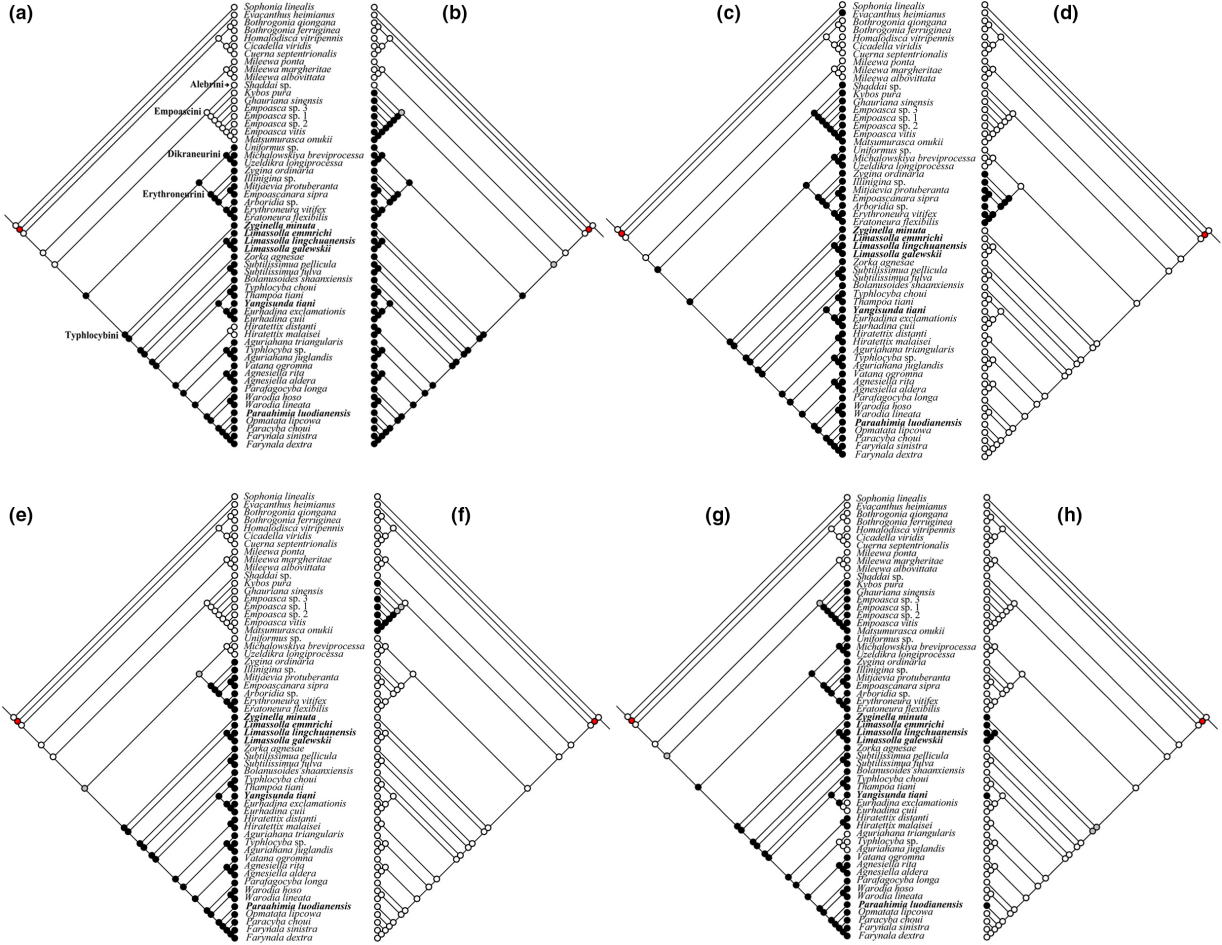
Ancestral character states reconstructions analysis based on ML tree (PCGs12_fna) using maximum‐likelihood method. The images at right illustrate seven morphological characters: a, ocelli (white ball, present; black ball, absent); b, forewing appendix (white ball, present; black ball, absent); c, forewing closed anteapical cells (white ball, present; black ball, absent); d, hind wing anal vein (white ball, bifurcated; black ball, not forked); e, hind wing with distal extension of CuA vein beyond submarginal vein (white ball, absent; black ball, present); f, hind wing CuA vein (white ball, bifurcated; black ball, not forked); g, hind wing RP and MP vein (white ball, connected by crossvein; black ball, confluent); h, hind wing submarginal vein apparently connected directly to CuA vein (white ball, absent; black ball, present). The gray balls indicate feature unknown. Taxa previously included in Zyginellini are indicated in bold

Eight typhlocybine morphological characters were traced over the BI consensus tree and ML tree (PCG12_fna). Overall, these reconstructions indicate that some characters used previously to diagnose tribes of Typhlocybinae exhibit considerable homoplasy (Figure 4; Figure S6). Ocelli were retained by Alebrini and Empoascini and lost once in the common ancestor of Dikraneurini, Erythroneurini, and Typhylocybini. However, a reversal to the ancestral state occurred in Typhylocybini corresponding to the genus *Hiratettix* (Figure 4a). The forewing appendix is present in most non‐typhlocybine leafhoppers but absent in all Typhlocybinae except Alebrini; thus Alebrini has traditionally been viewed as the most “primitive” group of Typhlocybinae (Figure 4b). However, the recovered sister‐group relationship between Alebrini and Empoascini suggests that the appendix may either lost independently in Empoascini and the remaining tribes or lost in the common ancestor of Typhlocybinae and regained in Alebrini. The bifurcation of hind wing vein CuA, shared with the outgroup, was lost independently in Empoascini and Typhlocybini (Figure 4f). The distal part of the hind wing submarginal vein (Figure 4e) was lost independently in Erythroneurini and Typhlocybini and hind wing veins RP and MA (Figure 4g) became confluent and separated multiple times independently within Typhlocybini. The direct connection of the hind wing submarginal vein to CuA directly, the trait traditionally used to diagnose Zyginellini as a distinct tribe was acquired independently in three different lineages of Typhlocybini (sensu lato; Figure 4h). Except for the single derivation of the latter “Zyginellini” trait in the analysis of Lu et al. (2021), whose dataset included only three representatives of this tribe, our results are consistent with this prior study.

### 3.4 Divergence time estimates

**FIGURE 5:**
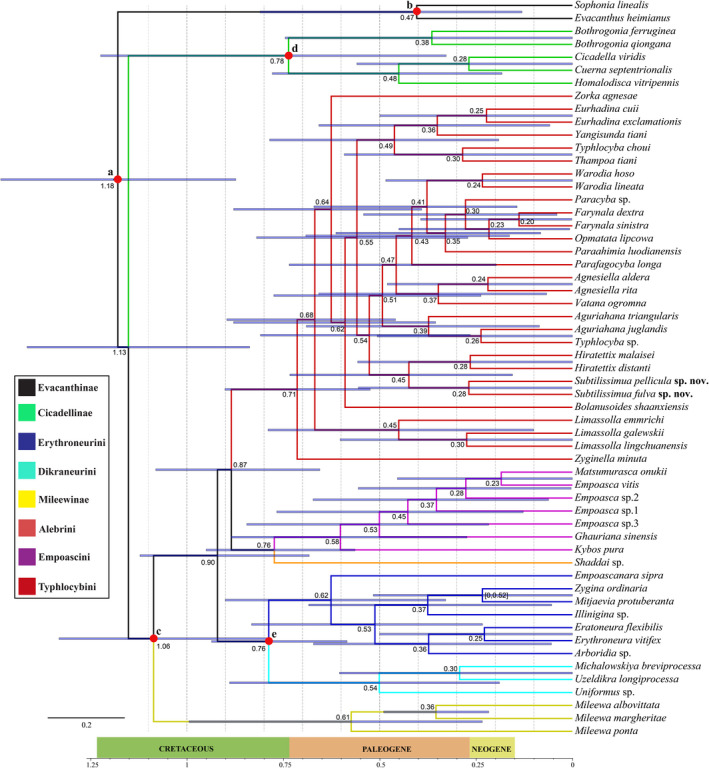
Chronogram showing divergence time estimates for major lineages of Typhlocybinae. Numbered nodes marked in red refer to fossil calibrations, a, root age, <174.1 MYA; b, 17.5–110 MYA; c, 44 MYA; d, 17.5–90 MYA; e, 17.5–90 MYA. Bars on nodes are the highest posterior density 95% confidence intervals (95% HPD). Scale: 1 = 100 MYA

A chronogram for Typhlocybinae divergence dates and topologies based on whole mitochondrial protein‐coding genes is shown in Figure 5. According to this result, Typhlocybinae arose ~118 MYA (94–142 MYA 95% HPD), during the middle Cretaceous period. Divergence of major lineages within Typhlocybinae was estimated to have begun ~99 MYA (78–119 MYA 95% HPD) with all tribes having first appeared during the Late Cretaceous. These date estimates are slightly younger than, but within the 95% confidence intervals, of those reported by Dietrich et al. (2017).

## 4 DISCUSSION

### 4.1 Mitogenome features

Most leafhopper mitochondrial genomes are highly conserved with an arrangement of the 37 genes consistent with that of the inferred ancestral insect, *Drosophila yakuba* (Clary & Wolstenholme, 1985). However, more than ten species in Cicadellinae, Deltocephalinae, Iassinae, Megophthalminae, and Ledrinae have the phenomenon of gene rearrangements with the previous studies (Du et al., 2017, 2019; Mao et al., 2017; Song et al., 2019; Wang et al., 2020) and our unpublished data. Only three types of gene rearrangements have been detected in Cicadellidae, which arise from three tRNA clusters: (1) in *Elymana* sp. (MK251130), *Osbornellus* sp. (MK251136), and *Stirellus bicolor* (MK251122), the tRNA cluster of trnA–R–N–S1–E–F is rearranged to trnR–E–F–A–N–S1; (2) in *Cofana unimaculata* (MK251095), the tRNA cluster of trnI–Q–M is rearranged to trnQ–I–M; (3) in *Cicadulina mbila* (MK251127), *Japananus hyalinus* (NC_036298), *Macrosteles quadrilineatus* (NC_034781), and *Macrosteles quadrimaculatus* (NC_039560), the tRNA cluster of trnW–C–Y is rearranged to trnY–W–C or trnC–W–Y. In this study, *Shaddai* sp. (MW284820) has the gene trnW translocated behind trnY, trnC–Y–W, with an intergenic spacer of 460 bp (Figure 1b). Gene rearrangements may represent an additional set of characters useful for phylogenetic reconstruction (Dowton et al., 2002; Tyagi et al., 2020) but additional data on leafhopper species will be needed to determine whether any such rearrangements are shared by closely related taxa.

### 4.2 Phylogeny, character evolution and tribal status

Our study indicates that mitogenome sequences are useful for resolving phylogenetic relationships of subfamily Typhlocybinae from the species to the tribal level. Analyses of both amino acid and nucleotide sequences yielded similar topologies with the main differences among analyses occurring among a few nodes that received low bootstrap support in one or more analysis. Our results agree with the result of a recent analysis by Lu et al. (2021) that incorporated partial sequence data from three nuclear and two mitochondrial genes in recovering Typhlocybinae as a well‐supported monophyletic group sister to Mileewinae (Mileewini). A comprehensive analysis of Membracoidea based on anchored hybrid data (Dietrich et al., 2017) failed to recover this relationship and the relationship of Typhlocybinae to other leafhopper subfamilies was poorly resolved by that study, so our results require further confirmation. Within Typhlocybinae, our results consistently supported the monophyly of the tribes Dikraneurini, Empoascini, Erythroneurini, and Typhlocybini (Alebrini was represented by a single species in our dataset).

**FIGURE 6:**
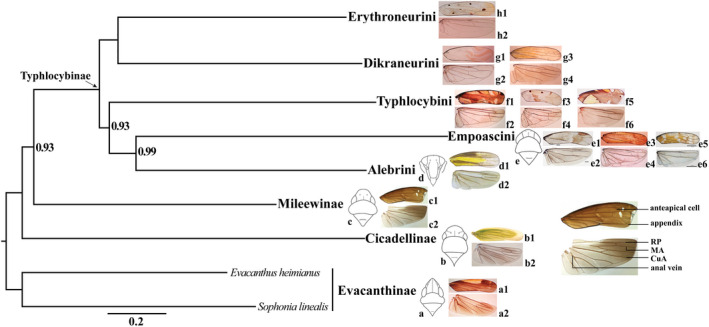
The ocelli and wings are mapped to the BI tree, separately. a–f, ocelli on crown; a1–h2, wings, photographed with transmitted light to highlight venation. Evacanthinae, a (followed Zhang, 2011), a1–a2, *Convexana albicarinata*; Cicadellidae, b, b1–b2, *Cicadella viridis*; Mileewinae, c, c1–c2, *Mileewa albovittata*. Typhlocybinae, Alebrini, d, d1–d2, *Shaddai* sp.; Empoascini, e, e3–e4 *Ghauriana sinensis*, e1–e2, *Joruma* sp. and e5–e6, *Heliona* sp. (followed, Xu, 2019); Typhlocybini, f1–f2, *Aguriahana triangularis*, f3–f4, *Agnesiella aldera*, f5–f6, *Zyginella minuta*; Dikraneurini, g1–g2, *Sweta bambusana*, g3–g4, *Dikraneura orientalis*; Erythroneurini, h1–h2, *Tautoneura formosa*

Lu et al. (2021) recovered the three species of Zyginellini included in their dataset as a monophyletic sister group of Typhlocybini but our larger sample of Zyginellini genera indicates that this tribe is polyphyletic. This supports the treatment of some recent authors (e.g., Dietrich, 2013; Hamilton, 1983) of Zyginellini as a junior synonym of Typhlocybini. Based on our results, the hind wing character traditionally used to separate Typhlocybini from Zyginellini (absence of the distal segment of vein CuA; Figure 6, f6) is homoplasious and has been derived independently in at least three unrelated lineages. Another hind wing character, veins RP and MA separate versus confluent (Figure 4, character g), previously used to distinguish Eupterygini from Typhlocybini but our results also indicate considerable homoplasy for this trait. Unfortunately, the extent of homoplasy in these characters remains uncertain because several relationships within Typhlocybini are unstable in our analyses as well as in the results of Lu et al. (2021). More detailed analyses including more characters and exemplar taxa are needed to provide an improved assessment of relationships among genera of Typhlocybini.

Details of relationships within individual tribes are difficult to compare between our results and those of Lu et al. (2021) given that the taxon samples of the two studies overlap only partially. Nevertheless, we note that several branches pertaining to relationships among genera of Typhlocybini, as well as a few branches within the other tribes, were extremely short and received low bootstrap support in both studies. This suggests that neither complete mitogenome data nor data from a few nuclear and mitochondrial genes will suffice to completely resolve typhlocybine phylogeny with high confidence, although increased taxon sampling may also help improve phylogenetic resolution in this group.

Our results are generally consistent with morphology‐based classifications proposed recently. Young (1965) moved Mileewini (as Mileewanini) to Typhlocybinae but this was rejected by Mahmood (1967). Young (1968) later suggested treating Mileewinae as a separate subfamily. Nevertheless, Mileewinae are similar to Typhlocybinae in having relatively small, slender bodies, reduced forewing venation, and slender hind tarsi (Dietrich, 2011). Previous phylogenetic studies have not reached a clear consensus on the sister‐group relationship of Typhlocybinae (Balme, 2007; Dietrich, 2013b; Dietrich et al., 2017; Wang et al., 2017). Our results are also equivocal in this regard. The ML trees from analysis of amino acid sequences of PCGs (Figure 2c) and the nucleotide sequences of PCGs and concatenated rRNAs (Figure 2e) recovered a sister‐group relationship between Typhlocybinae and Cicadellinae (sensu stricto), but all other analyses (Figure 2a,b,d) and the tree topology tests (Table 2) consistently support Typhlocybinae as sister to Mileewinae. Considering the morphological similarities shared by Typhlocybinae and Mileewinae, the latter hypothesis seems to be more plausible.

The tribal relationships in Typhlocybinae have long been controversial (Balme, 2007; Dietrich et al., 2017; Mahmood & Ahmed, 1968; Wagner, 1951; Zhang, 1990). Our analyses consistently support a sister‐group relationship between Alebrini and Empoascini, as well as between Erythroneurini and Dikraneurini, in agreement with other recent analyses (Dietrich et al., 2017; Lu et al., 2021). The former relationship is also consistent with Wagner's intuitive morphology‐based hypothesis (1951), but not with those of Mahmood and Ahmed (1968) and Zhang (1990).

The main areas of uncertainty are the relationships between Typhlocybini and other tribes, and relationships among several deep nodes within Typhlocybini. Different analyses recovered Typhlocybini either as sister to Alebrini +Empoascini (Figure 2a,b) or as sister to Erythroneurini +Dikraneurini (Figure 2c,d,e), thus this relationship remains equivocal. Detailed morphology‐based cladistic analyses of Typhlocybinae have not been attempted but there appears to be some morphological support for the latter hypothesis (Typhlocybini+(Dikraneurini+Erythroneurini)). For example, all Erythroneurini and most Typhlocybini (except a few Neotropical taxa) have the hind wing submarginal vein absent at the wing apex. The male subgenital plates of Typhlocybini, Dikraneurini, and Erythroneurini also have relatively few macrosetae compared to those of Alebrini and Empoascini. These traits are potential synapomorphies supporting the sister relationship of Typhlocybini to Dikraneurini+Erythroneurini recovered in most of our analysis as well as the analyses of Lu et al. (2021) and Dietrich et al. (2017).

Ancestral state reconstructions of key morphological characters previously used to define and diagnose tribes within Typhlocybinae (Dworakowska, 1979, 1993; Evans, 1963, 1971; Hamilton, 1983; Zhang, 1990; Table 3) indicate that some wing characters traditionally used to diagnose tribes are highly stable. Our analyses generally support the monophyly of tribes Empoascini, Dikraneurini, Erythroneurini, and Typhlocybini as defined morphologically by most authors. Thus, our analyses suggest that the wing vein characters traditionally used to define these groups are reliable. Nevertheless, our results also indicate that some morphological characters have undergone homoplastic changes during the evolution of the group. Ocelli may be present or absent within Empoascini and Typhlocybini. In Empoascini, all genera have ocelli, except *Beamerana* and *Paulomanus*. The latter genera, which have hind wing venation identical to that of many Empoascini, nevertheless grouped with Typhlocybini in a recent morphology‐based phylogeny and were tentatively placed in the latter tribe (Xu et al., 2021). Few genera in Typhlocybini have ocelli, for example, *Hiratettix* and *Caknesia*, but we were able to include only the former in our analyses and it occupies a relatively derived position within the tribe, suggesting that ocelli were lost and regained at least once in this tribe (Figure 4a). Loss of the hind wing submarginal vein is also apparently homoplastic with independent losses apparently occurring in Erythroneurini and Typhlocybini (Figure 4e). Hind wing veins RP and MA have also apparently become confluent in the common ancestor of Erythroneurini and Dikraneurini and independently in Empoascini and Typhlocybini (Table 3). Loss of the branched hind wing anal vein occurs only in the Dikraneurini +Erythroneurini lineage but further analyses with a larger sample of taxa will be needed to determine the extent of homoplasy in this character.

**TABLE 3:** Comparison of tribal characters states in Typhlocybinae

Feature	Ocelli	Forewing appendix	Hind wing anal vein	Hind wing submarginal vein	Hind wing CuA vein	Hind wing RP and MP vein
Tribe						
Alebrini	+	+	+	+	+	+
Empoascini	+,–	–	+	+	+, –	+, –
Typhlocybini	+, –	–	+	–	–	+, –
Dikraneurini	–	–	+, –	+, –	+, –	–
Erythroneurini	–	–	–	–	+	–

Note

The symbols “+” and “–” indicated the following morphological characters. Ocelli and forewing appendix: “+,” present; “–,” absent. Hind wing anal vein: “+,” bifurcated; “–,” not forked. Hind wing submarginal vein: “+” extended beyond CuA vein; “–” not extended beyond CuA vein. Hind wing CuA vein: “+,” bifurcated; “–,” not forked; Hing wing RP and MP vein: “+,” connected by crossvein; “–,” confluent. The symbol “+, –” denotes the presence of both character states.

The molecular divergence time estimates indicate that Typhlocybinae and extant tribes originated during the Middle and Late Cretaceous, respectively. Thus, diversification of major lineages in this group seems to have roughly coincided with the diversification of angiosperms during the Cretaceous (Foster et al., 2016; Hamilton, 1992, 1994; Ledyard, 1974). Our divergence time estimates for tribes of Typhlocybinae are considerably younger than those reported for the same branches by Dietrich et al. (2017), possibly due to the denser taxon sampling of our study. Thus, in our study, the splits between Erythroneurini and Dikraneurini, and between Alebrini and Empoascini are both estimated at 76 MYA, compared to the 95 and 112 MYA, respectively, reported by Dietrich et al. (2017). Ninety‐five percent confidence intervals of both studies are wide and broadly overlap, indicating that the available methods and data are only able to provide approximate estimates of the times of origin of these major leafhopper lineages. Future analyses incorporating additional fossil evidence (unavailable at present), diversified clock models, dating methods, and rate priors may yield improved estimates.

### 4.3 Species misidentifications

Song et al. (2017) analyzed relationships within Cicadomorpha using mitochondrial genome data, including a species identified as “*Typhlocyba* sp.” (GenBank accession number, KY039138). In our phylogenetic results this sample did not group with *Typhlocyba choui*, but instead grouped with the genus *Aguriahana* with high support in all phylogenetic trees (PP = 1, SH‒aLRT = 100, UFB = 100; Figure 1). In addition, the COX1 *p*‐distance between “*Typhlocyba* sp.” (GenBank accession number, KY039138) and *Typhlocyba choui* was 18%, but the former was only 1.2% divergent from *Aguriahana juglandis* (Table S3). Based on the criteria of intraspecific genetic distance from 0% to 2% (Hebert et al., 2003), we suggest that “*Typhlocyba* sp.” (GenBank accession number, KY039138) should be defined a species of *Aguriahana*.

## 5 CONCLUSIONS

This study sequenced 28 typhlocybine mitogenomes, representing all currently recognized tribes. We report the first known mitochondrial gene rearrangement within Typhlocybinae (Alebrini). Despite some variability among phylogenetic estimates based on different datasets, the results consistently support the monophyly of Typhlocybinae and four tribes for which multiple representatives were included. In contrast to another recent analysis (Lu et al., 2021), Zyginellini was consistently polyphyletic in our results. Our results support the sister‐group relationship of Typhlocybinae to Mileewinae but this relationship received only moderate branch support. Ancestral character state reconstructions (ACSR) suggest that some morphological characters traditionally considered important for diagnosing tribes are homoplastic. A key to tribes of Typhlocybinae is provided and new taxonomic changes are proposed based on the phylogenetic results, morphology and genetic distances. Although mitogenome sequence data appear to be broadly informative of relationships at various levels in the taxonomic hierarchy of Typhlocybinae, further study incorporating a larger taxon sampling and additional morphological and molecular evidence (e.g., nuclear genes, anchored hybrid loci) should help further clarify the phylogeny of this highly diverse group.

## TAXONOMY

### Key to tribes of Typhlocybinae

1. Forewing appendix present (Figure 6, d1) …………………….…**Alebrini**

–. Forewing appendix absent …………………………………………...……..2

2. First forewing apical cell shorter than one third length of forewing (Figure 6, e1, e3, e5, f1, f3, f5)……………………………………………...…3

–. First forewing apical cell longer than one third length of forewing (Figure 6, g1, g3, h1)…….………………………………………………………..4

3. Hind wing submarginal vein extended beyond CuA vein but not surpassing MA vein or “MA + RP” vein (Figure 6, e2, e4, e6)……….………………………………………….………………………………….………**Empoascini**

–. Hind wing submarginal vein not extended beyond CuA vein (Figure 6, f2, f4, f6)..………………………………………………………**Typhlocybini**

4. Hind wing submarginal vein complete, extending along costal margin and connected to apices of all longitudinal veins (Figure 6, g2, g4)..……………….……………………….……………………………...…... **Dikraneurini**

–. Hind wing submarginal vein shorter than CuA vein (Figure 6, h2).…………..………………………………………………………………**Erythroneurini**

### Typhlocybini Kirschbaum, 1868

*Subtilissimia* Yan & Yang gen. nov.

http://zoobank.org:act:262A48A8‐86C3‐4B02‐8EB6‐5C4726AF13D2

**Type species:** *Subtilissimia fulva* Yan & Yang sp. nov.

**FIGURE 7:**
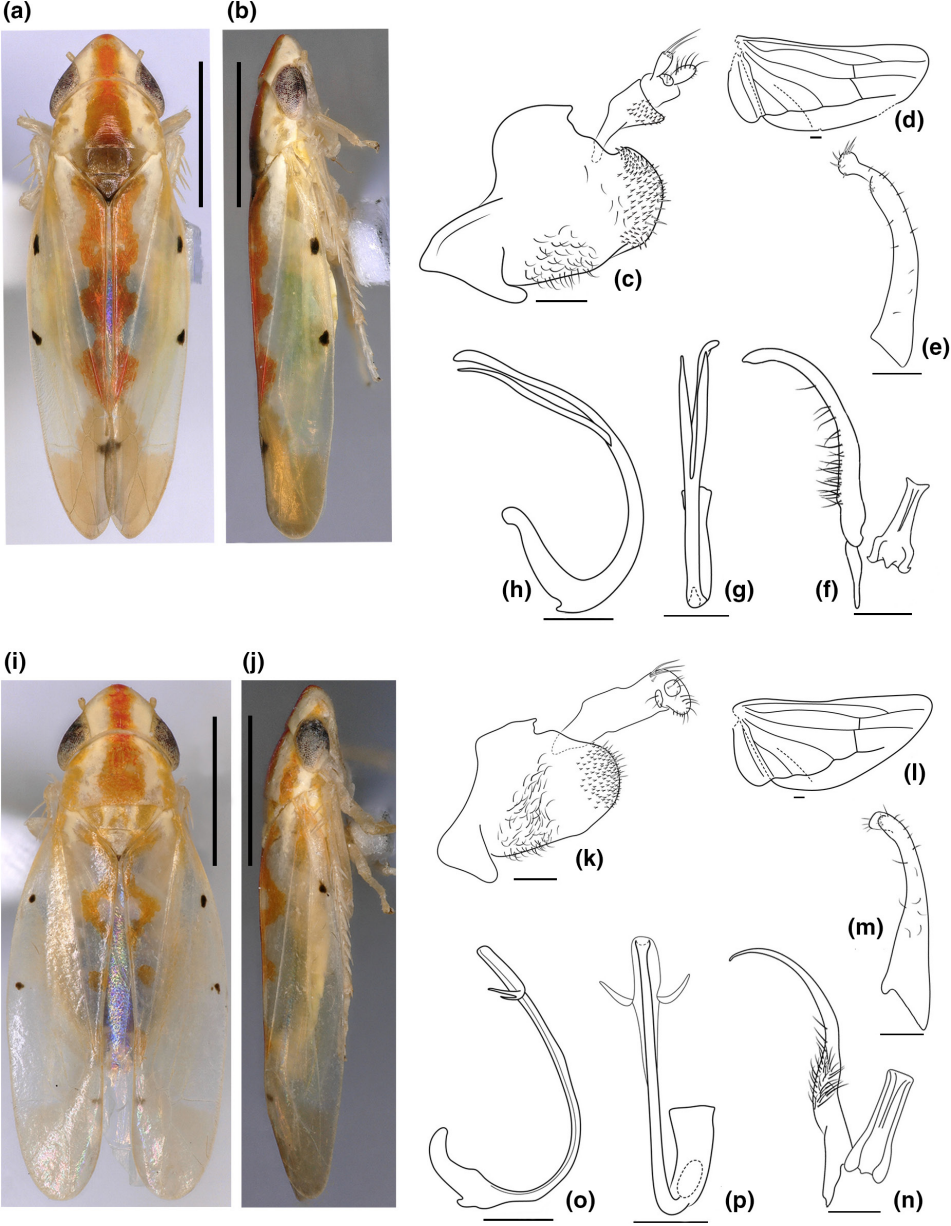
a–h, *Subtilissimia fulva* Yan & Yang sp. nov. a, habitus, dorsal view; b, habitus, lateral view; c, male genitalia, lateral view; d, hind wing; e, subgenital plate, ventral view; f, style and connective, ventral view; g, aedeagus, lateral view; h, aedeagus, ventral view. i–p, *Subtilissimia pellicula* Yan & Yang sp. nov. i, habitus, dorsal view; j, habitus, lateral view; k, male genitalia, lateral view; l, hind wing; m, subgenital plate, ventral view; n, style and connective, ventral view; o, aedeagus, lateral view; p, aedeagus, ventral view

**Diagnosis.** The new genus differs from other genera in this tribe by the following combined characters: small to medium sized, depressed, and ovoid (Figure 7a,b,i,j); head with a longitudinal reddish broad stripe from crown apex to posterior margin of pronotum; subgenital plate without macrosetae near base; style with numerous microsetae in middle part (outer margin); connective Y‐shaped; aedeagus dorsal apodeme well developed, without process on base.

**Description.** Length of male 3.5 to 3.7 mm, female 3.5 to 3.6 mm. Dorsum brown‐yellowish; head with longitudinal reddish broad stripe from crown apex to posterior margin of pronotum. Forewing semitransparent; veins pale white; apical cells infuscated; forewing clavus with reddish stripe, wave‐shape; brochosome area with two small dark spots; MP’’+CuA’ vein with black spot on apex (Figure 7a,b,i,j). Face yellow. Body flat in lateral view (Figure 7b,j); crown produced; coronal suture distinct, not extended beyond the midline length of crown (Figure 7a), or indistinct (Figure 7b); Head subequal to pronotum width, length of crown at midline slightly longer than interocular width; scutellar suture distinct, nearly reach to sides; forewing apex rounded, the 2nd apical cell subequal to 4th apical cell; the 3rd apical cell smallest, triangular; hind wing R and MP veins directly connected at apex (Figure 7d,l).

**Male genitalia.** Pygofer side with posterior margin rounded and with two clusters of setae on posterior and ventral parts; without macrosetae and process (Figure 7c,k); subgenital plate gradually narrowed to apex, without big macrosetae at base; apex forked, or not forked (Figure 7c,k); style slender, with a dense coverage of microsetae on outer margin; connective Y‐shaped, with central lobe developed (Figure 7f,n); aedeagus with dorsal apodeme well developed, preatrium weak; shaft slender, with pair of symmetrical processes; phallotreme apical (Figure 7h,o).

**Remarks**.

The new genus is similar to *Typhlocyba* in coloration and male genitalia but can be distinguished from the latter by the absence of basal macrosetae on the subgenital plate and presence of dense microsetae at the midlength of the paramere.

**Etymology.** This generic name is feminine, formed by the Latin word “*Subtilissimus*” which means “slender or fine,” referring to its aedeagal shaft in lateral view.

**Distribution.** China (Yunnan)

*Subtilissimia fulva* Yan & Yang sp. nov.

http://zoobank.org:act:BFCDAE7D‐C5E4‐4896‐A841‐E3BA4A559DE3

Figure 7a–h

**Material examined.** Holotype, ♂, Mengla County, Yunnan Province, 608 m, 12 May 2015, Coll. Bin Yan. Paratypes, 1♂, 3♀♀, same data as holotype; 1♂, Mengla County, Yunnan Province, 604 m, 16 Nov. 2018, Coll. Likun Zhong.

**Description.** Length of male 3.5 to 3.7 mm. Coronal suture indistinct (Figure 7a). Forewing clavus with deeply reddish stripe, elongated to apex (Figure 7a,b). Male pygofer side slightly convex on upper posterior margin (Figure 7c); style slender, parallel‐sided (Figure 7f); aedeagal shaft tubular, with pair of lateral processes arising near mid length, extended distally and slightly divergent from shaft, almost reaching shaft apex (Figure 7g,h).

**Etymology.** The species name was derived from the Latin adjective “fulvus” = reddish‐yellow, tawny, amber‐colored, which refers to the coloration of the longitudinal patch on the crown.

**Remarks**.

The new species is very similar to *Subtilissimia pellicula* sp. nov. in size and coloration, but differs in having the aedeagal processes arising near the shaft mid length, longer, and not forked near the apex in lateral view (Figure 7g,h).

**Distribution.** China (Yunnan)

*Subtilissimia pellicula* Yan & Yang sp. nov.

http://zoobank.org:act:2B04D9D2‐665D‐4A6D‐B9C9‐E75F3171D144

Figure 7i–p

**Material examined.** Holotype, ♂, Simian Mountain, Chongqing, 1100 m, 12 Sep. 2017, Coll. Bin Yan. Paratypes, 3♂♂, same data as holotype.

**Description.** Body length 3.5 to 3.6 mm. Coronal suture distinct, short (Figure 7i). Forewing with reddish stripe on hind margin, not reaching clavus apex (Figure 7i,j). Male pygofer side rounded on posterior margin (Figure 7m); style slender, apex sharp, and slightly curved (Figure 7n); Aedeagus tubular in ventral view, with membranous sides; shaft with pair of dorsal processes arising near apex, curved dorsally, forked near base (Figure 7o,p).

**Etymology.** The species name was derived from the Latin noun “*pellicula*” = “small skin”, which refers to a membranous structure on the shaft. It should be treated as a noun in apposition.

**Remarks**.

The new species is very similar to *Subtilissimia fulva* sp. nov. in size and coloration, but differs in having the aedeagal processes arising near the shaft apex, curved dorsally, and forked near the apex in lateral view (Figure 7o,p).

**Distribution.** China (Chongqing)

### *Farynala* Dworakowska, 1970

*Farynala dextra* Yan & Yang, 2017

*Farynala dextra* Yan & Yang, 2017: 520.

*Farynala sinistra* Yan & Yang, 2017: 520 syn. nov.

**Note.** Yan and Yang (2017) described the species of *Farynala sinistra* and *Farynala dextra*, distinguishing them according to numbers of macrosetae on the pygofer side and the orientation of the aedeagal processes being opposite such that they appear to be mirror images of one another. Our current phylogenetic results (Figures S1‒S5) and the small genetic distance between these two taxa based on COX1 *p*‒distance = .51% strongly indicates that these taxa are conspecific. Thus, the two species names are here treated as synonyms.

## AUTHOR CONTRIBUTIONS

**Bin Yan:** Formal analysis (lead); Investigation (equal); Methodology (lead); Writing – original draft (lead); Writing – review & editing (equal). **Christopher H. Dietrich:** Conceptualization (equal); Methodology (equal); Writing – review & editing (lead). **Xiaofei Yu:** Formal analysis (equal); Investigation (equal); Methodology (equal). **Meng Jiao:** Data curation (equal); Formal analysis (equal); Investigation (equal). **Renhuai Dai:** Formal analysis (equal); Investigation (equal). **Maofa Yang:** Conceptualization (equal); Funding acquisition (lead); Project administration (equal); Writing – review & editing (equal).

## CONFLICTS OF INTEREST

The authors declare no conflict of interest to disclose.

## Supporting information

Fig S1

Fig S2

Fig S3

Fig S4

Fig S5

Fig S6

Table S1‐S5

## Data Availability

The molecular datasets used for all analyses are available in the Illinois Data Bank (https://doi.org/10.13012/B2IDB‐8761342_V1). GenBank accession numbers: MW264489, MW272457, MW272458, MW284820–MW284843.
