# Supplementary figures and images for: Mitogenomic phylogeny of Typhlocybinae (Hemiptera: Cicadellidae) reveals homoplasy in tribal diagnostic morphological traits
Source: Ecol Evol. 2022 Jun 6;12(6):e8982. doi: 10.1002/ece3.8982 (PMC9170537; doi:10.1002/ece3.8982)

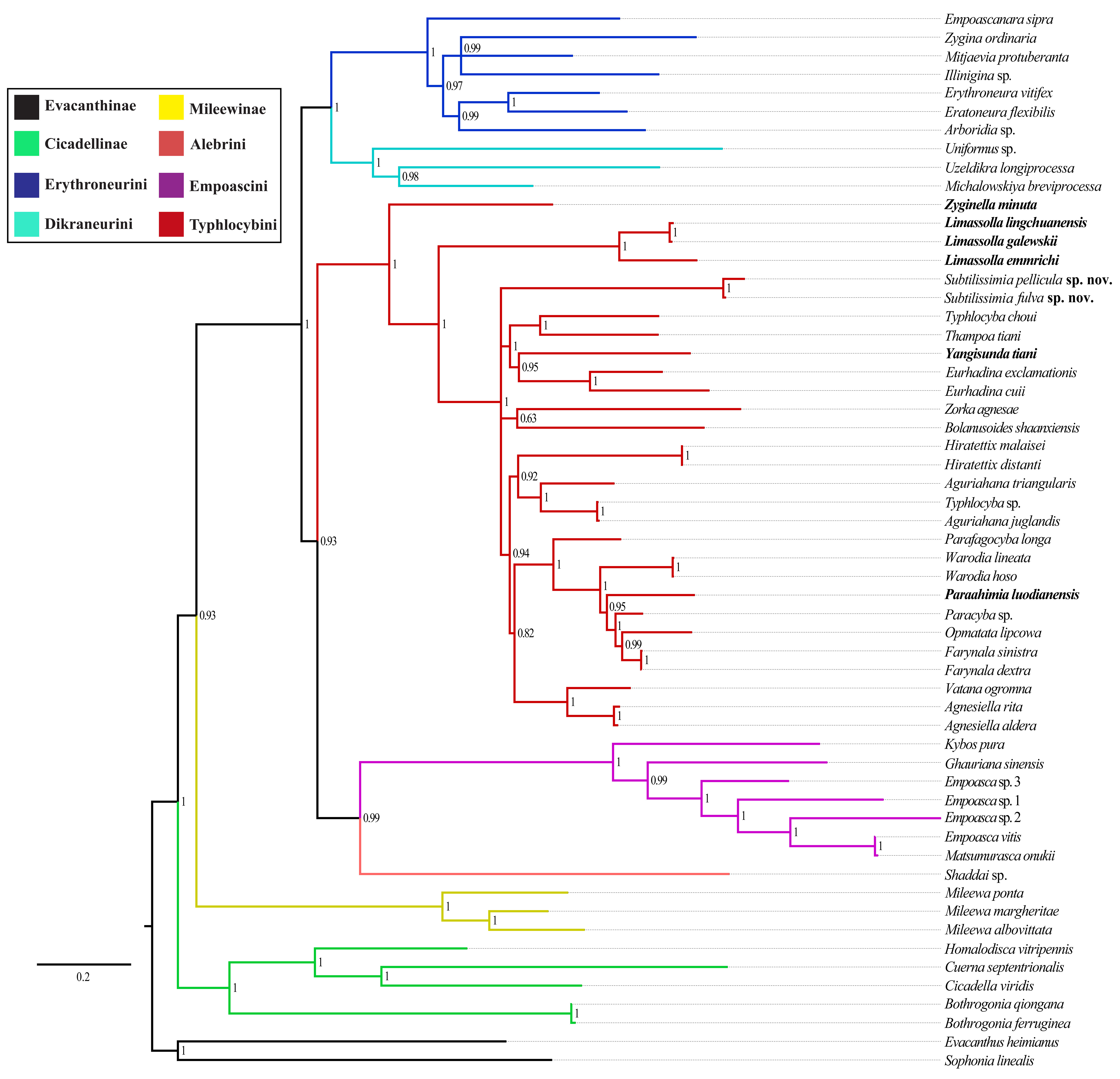

Supplement: Supplementary file 1 — Fig S1 [file ECE3-12-e8982-s001.jpg]

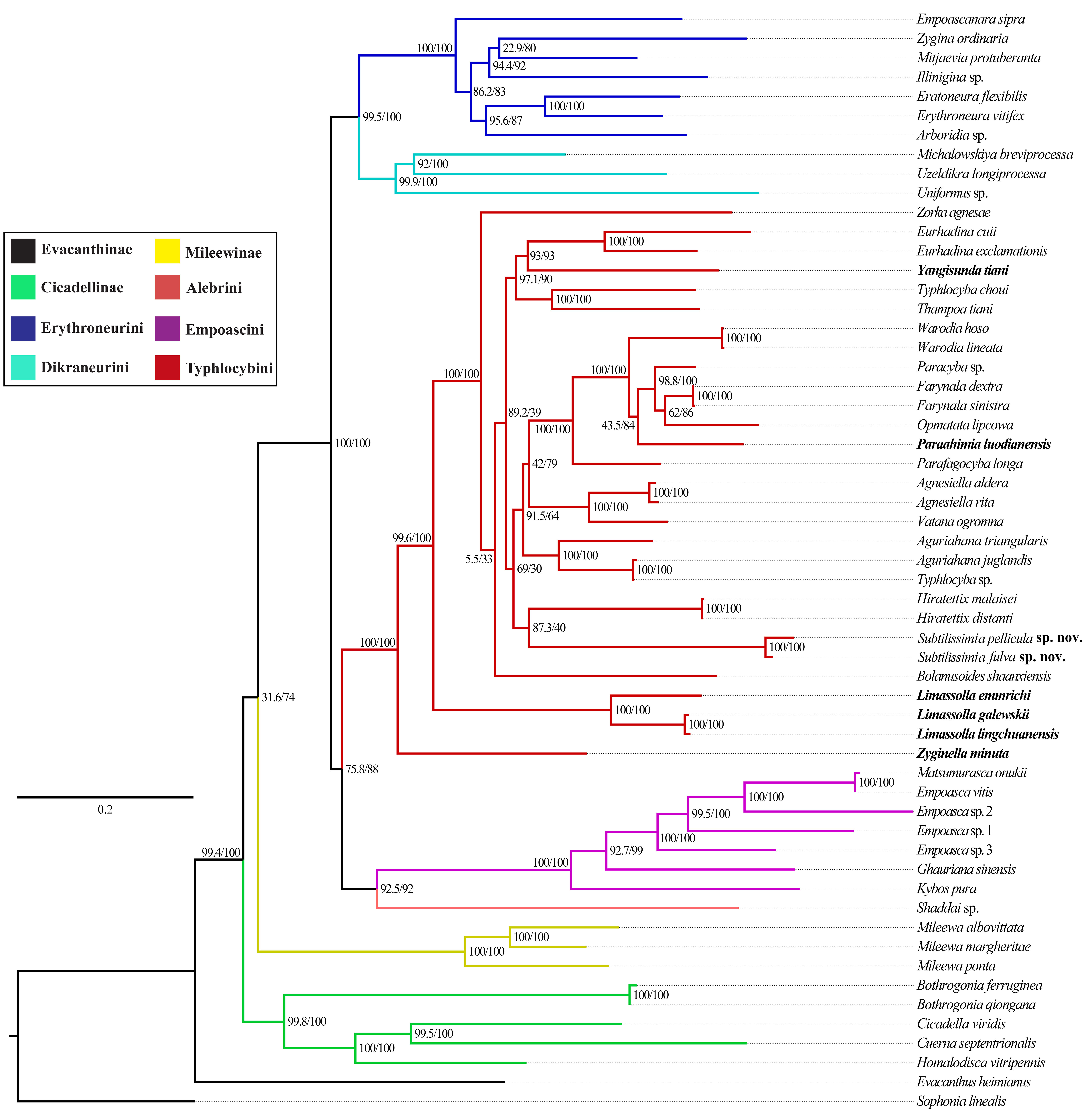

Supplement: Supplementary file 2 — Fig S2 [file ECE3-12-e8982-s003.jpg]

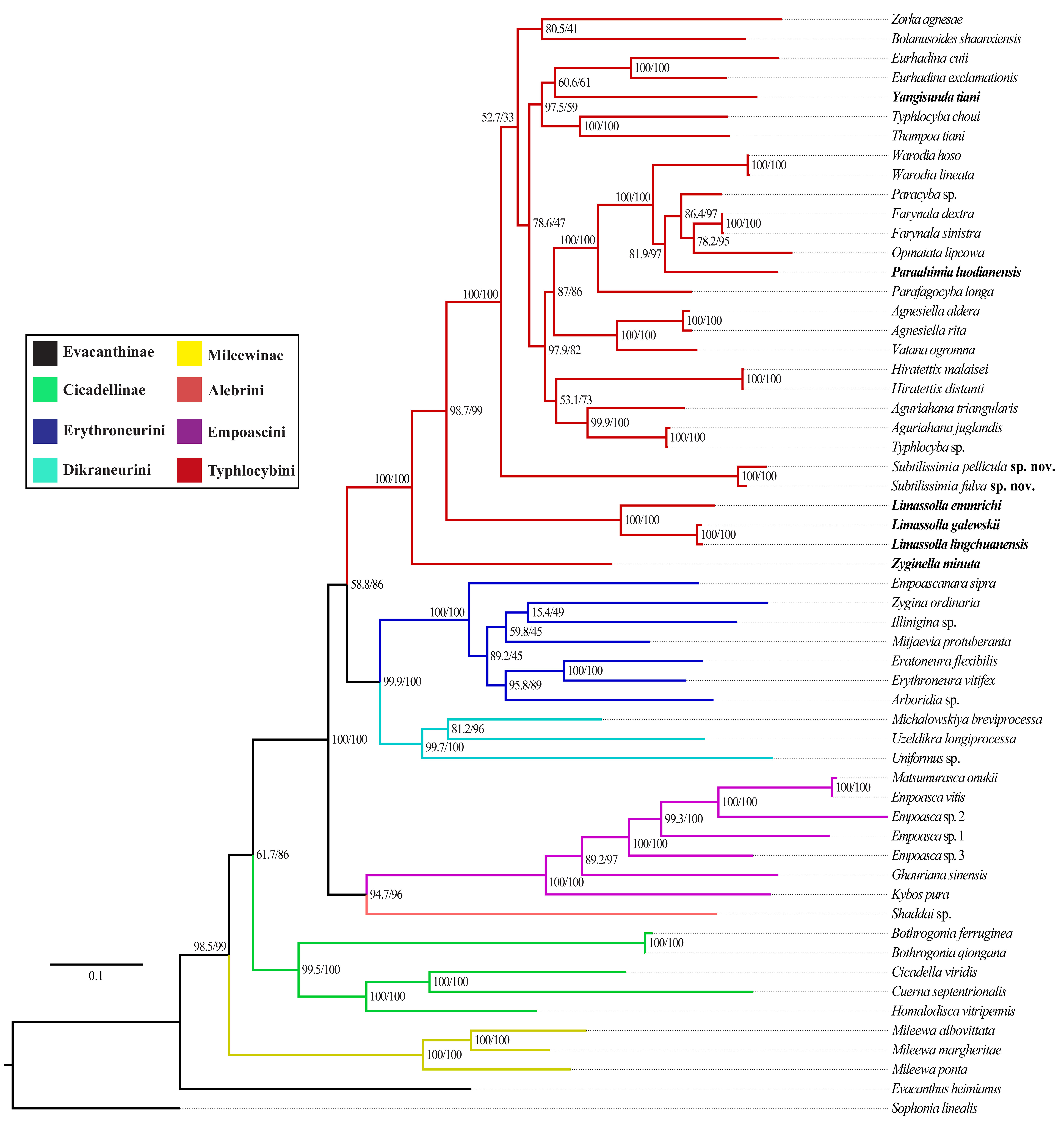

Supplement: Supplementary file 3 — Fig S3 [file ECE3-12-e8982-s002.jpg]

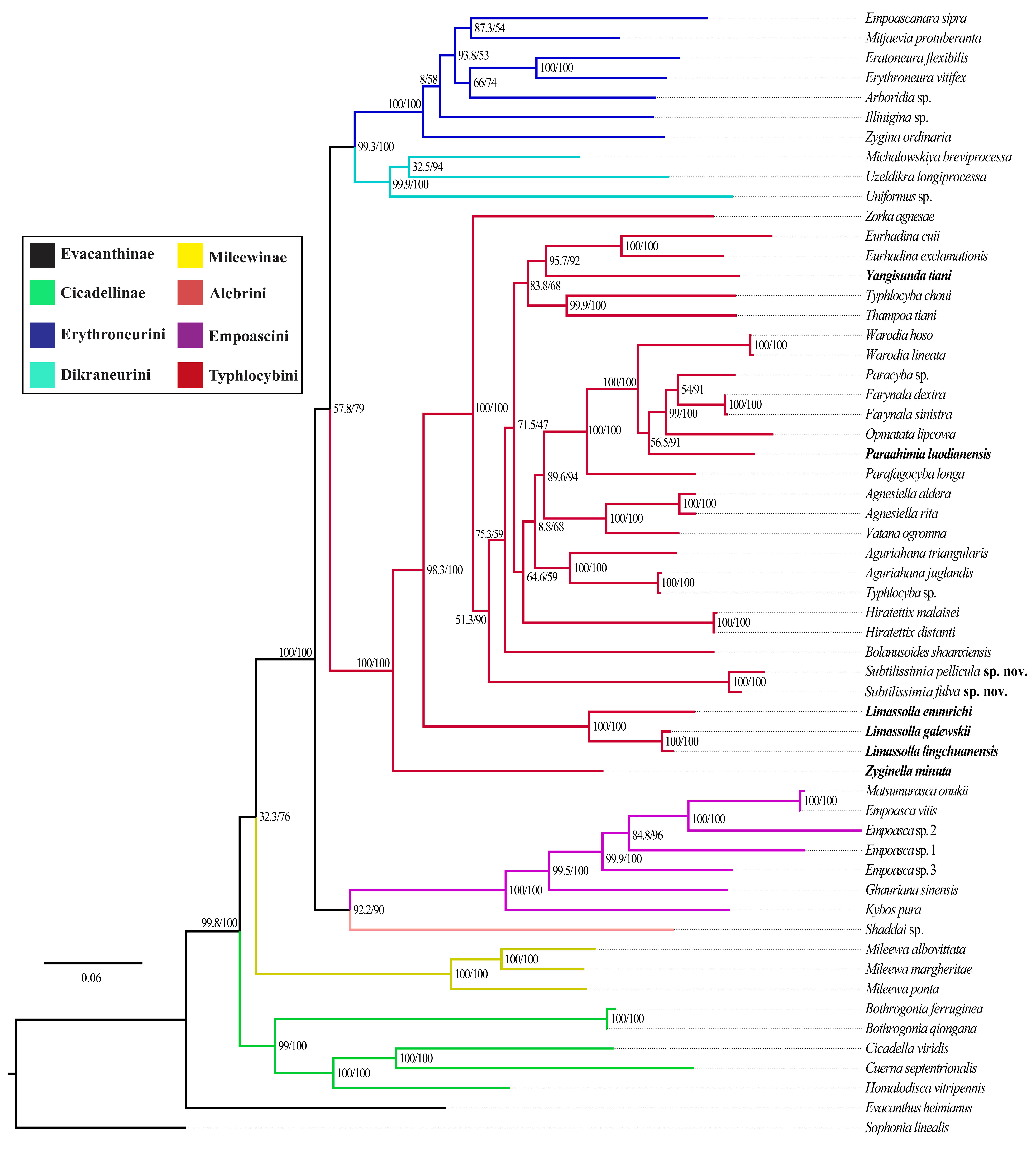

Supplement: Supplementary file 4 — Fig S4 [file ECE3-12-e8982-s006.jpg]

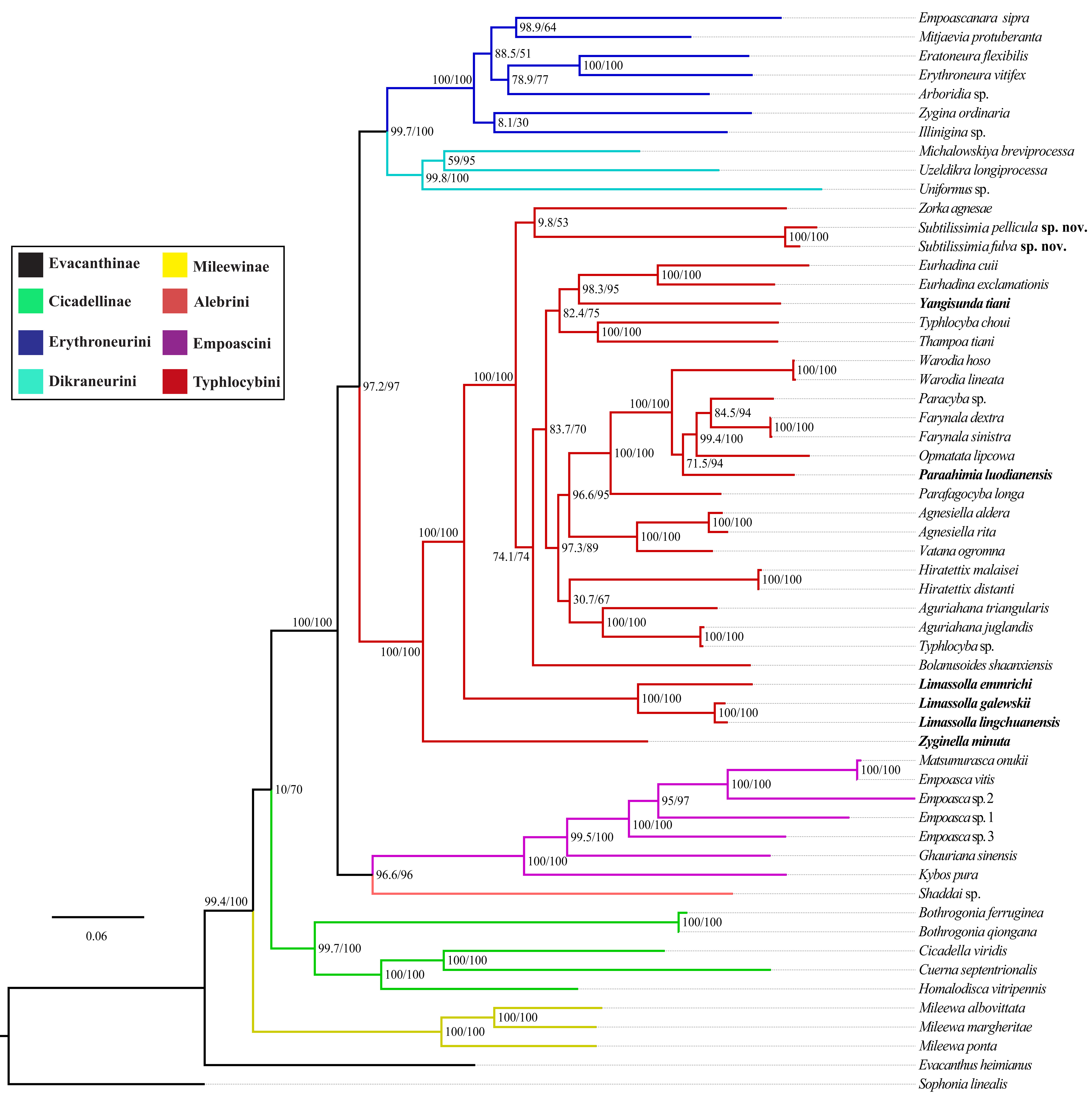

Supplement: Supplementary file 5 — Fig S5 [file ECE3-12-e8982-s007.jpg]

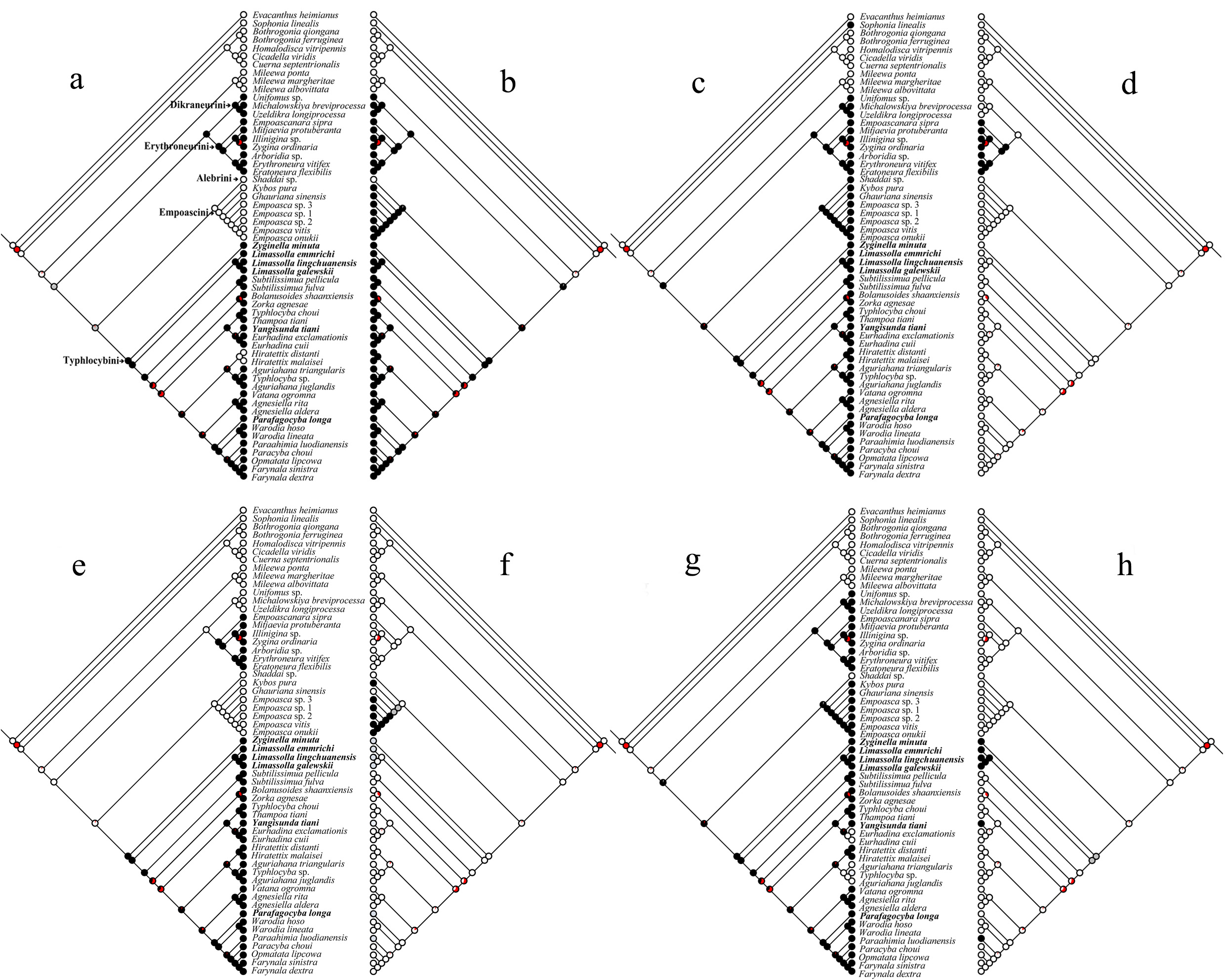

Supplement: Supplementary file 6 — Fig S6 [file ECE3-12-e8982-s005.jpg]
